# N-acetylcysteine may improve endometrial receptivity by modulating endometrial stromal cells in goats

**DOI:** 10.5194/aab-67-361-2024

**Published:** 2024-07-17

**Authors:** Kaibin Fu, Xiang Chen, Xingzhou Tian, Wen Tang, Ting Gong, Yan Zhang, Taotao Ji

**Affiliations:** 1 Key Laboratory of Animal Genetics, Breeding and Reproduction in the Plateau Mountainous Region, Ministry of Education, Guizhou University, Guiyang 550025, China; 2 College of Animal Science, Guizhou University, Guiyang 550025, China; 3 Liupanshui City Animal Husbandry and Aquaculture Development Center, Liupanshui 553000, China

## Abstract

Endometrial receptivity is essential for successful embryo implantation and pregnancy maintenance, and the achievement of normal physiological function of endometrial stromal cells (ESCs) is an important factor in the establishment of receptivity. N-acetylcysteine (NAC) was found to be beneficial as a small-molecule antioxidant for improving animal reproductive performance, but it is unclear whether NAC can improve receptivity by modulating ESCs in goats. In this study, we successfully isolated and cultured the ESCs of Qianbei Ma goats, used different concentrations of NAC to culture the ESCs of Qianbei Ma goats, and explored the effects of NAC on the biological functions of the ESCs of Qianbei Ma goats by using CCK-8, qRT-PCR, ELISA and flow cytometry. The results showed that 200 µ mol L ${}^{-1}$ NAC may promote the proliferation of ESCs in Qianbei Ma goats by increasing the expression of *PCNA*, *Cyclin D2* ( P<0.01 ) and *Cyclin E* ( P<0.05 ) mRNAs. Meanwhile, 200 µ mol L ${}^{-1}$ NAC increased the cell viability ( P<0.01 ) and enhanced the superoxide dismutase (SOD) and glutathione reductase (GR) activities ( P<0.01 ) of ESCs in Qianbei Ma goats. In addition, 200 µ mol L ${}^{-1}$ NAC increased the mitochondrial membrane potential of ESCs ( P<0.01 ). Notably, 200 µ mol L ${}^{-1}$ NAC promoted the expression of the mRNA of the endometrial receptivity marker genes *HOXA10*, *PTGS2*, *PGR* ( P<0.01 ) and *LIF* ( P<0.05 ). Thus, our results suggest that NAC may benefit the establishment of endometrial receptivity in goats by regulating the proliferation, antioxidant properties, mitochondrial membrane potential and expression of endometrial receptivity marker genes in goat ESCs.

## 1 Introduction

Early embryo loss is an important factor affecting the reproductive efficiency of goats, and it not only causes huge economic losses but also leads to increased culling of breeding stock (Jonker, 2004). One study found that embryo–uterus asynchrony is a key factor in the failure of embryos to successfully implant (Mondal et al., 2015). Successful implantation of an embryo into the maternal uterus is a critical step in establishing pregnancy in mammals (Vasquez and DeMayo, 2013; Matsumoto, 2017). The quality of the embryo and endometrial receptivity are the two main factors affecting successful embryo implantation (Jain et al., 2022). Endometrial receptivity is the ability of the endometrium to allow normal implantation of the embryo, optimal receptivity plays a crucial role in successful embryo implantation (Berg et al., 2010; Wiltbank et al., 2016), and existing studies have shown that endometrial receptivity is regulated by a combination of factors, including the interconnection of endometrial stromal cells (ESCs) and endometrial epithelial cells (EECs), the synergistic effects of related hormones such as E2 and P4 as well as changes in the expression levels of related genes (Teh et al., 2016). Notably, the proliferation, differentiation and apoptosis of ESCs affect the establishment of endometrial receptivity (Tu et al., 2014). One study showed that two-thirds of embryo implantation failures are due to endometrial receptivity damage, which does not provide good conditions for embryo implantation and growth (Lédée-Bataille et al., 2002). Therefore, it is important to improve endometrial receptivity during embryo attachment. Some studies found that antioxidants are beneficial in improving endometrial receptivity (Chuffa et al., 2019; Noh et al., 2020; Corachán et al., 2021).

N-acetylcysteine (NAC), a natural derivative of L-cysteine and a precursor of reduced glutathione (GSH), is a small-molecule antioxidant that scavenges free radicals to achieve the body's antioxidant effects (Mokhtari et al., 2017; Aldini et al., 2018). NAC attenuates lipopolysaccharide (LSP)-induced cytotoxicity by inhibiting oxidative stress and mitochondrial dysfunction (Raza et al., 2014). In addition, a study on human apical cells found that NAC reduced LSP-induced inflammation levels and maintained mitochondrial activity (Jariyamana et al., 2021). Oral NAC supplementation can improve sperm count and viability in infertile men, increase the antioxidant capacity of sperm, and reduce the proportion of abnormal sperm morphology, DNA fragmentation and protamine deficiency (Jannatifar et al., 2019). NAC can reduce perfluorooctanoic-acid-mediated oxidative stress (Owumi et al., 2021) and inflammatory responses to enhance reproductive function in rats and restore reproductive defects caused by γ -glutamyl-transpeptidase deficiency in mice (Wang et al., 2021). For the ovary, NAC can maintain the quality of mouse oocytes, delay the aging of oocytes (Wang et al., 2019), and restore the fertility of vitrified oocytes (Mukunoki et al., 2019) and the function of oocyte meiosis under heat stress conditions (Hu et al., 2020). In addition, during cryopreservation of ovarian follicles, oocytes and embryos, NAC can achieve antioxidant effects by improving mitochondrial function, cell viability and apoptosis and by reducing the production of reactive oxygen species (ROS) (Li et al., 2019; Barrozo et al., 2021). NAC can also promote normal embryonic development by increasing GSH biosynthesis and regulating cell proliferation (Truong and Gardner, 2017; Rastogi et al., 2019). It was found that NAC supplementation during late gestation in sows could alleviate maternal–placental oxidative stress and inflammatory response, improve placental function (Luo et al., 2019), affect placental trophoblast differentiation, and regulate placental function by regulating the synthesis of steroids in placental trophoblast cells of sows, promoting cell proliferation and inhibiting apoptosis (Ding et al., 2021). In our previous study, we showed that NAC could increase the number of lambs born in Nubian goats; promote NO production; improve the anti-inflammatory pathway of the endometrium (Luo et al., 2021); increase the serum levels of FSH, LH, E2, P4, superoxide dismutase (SOD) and GSH-Px in Qianbei Ma goats (Yang et al., 2022); and activate or inhibit the related signaling pathways, thus affecting the reproductive performance of goats (Fu et al., 2022). This shows that NAC has a positive effect on the regulation of animal reproductive performance, but few studies have been reported on the mechanism of NAC regulation of endometrium in goats. Studies have shown that ESCs are essential for the establishment of endometrial receptivity (Egashira and Hirota, 2013; Lucas et al., 2016). Therefore, we hypothesized that NAC could positively modulate the physiological function of ESCs in goats, thereby improving their endometrial receptivity. Based on this hypothesis, we added NAC to the cell culture medium with Qianbei Ma goat ESCs to investigate the effects of NAC on the proliferation, gene expression and antioxidant function of goat ESCs and to provide basic data to study the molecular regulatory mechanism of NAC affecting the reproductive function of goats.

## 2 Materials and methods

### 2.1 Isolation, culture and identification of goat ESCs

ESCs of goats were collected from the healthy uteruses of three 36-month old non-pregnant Qianbei Ma goats (Fuxing Herd Co., Ltd., Guizhou, China). The uteruses of the goats were collected rapidly after slaughter and were first washed with saline and then with phosphate buffer solution (PBS) containing penicillin-streptomycin (Xavier Biotechnology Co., Ltd., Wuhan, China). Finally, the uterine horns of the goats were used to extract the ESCs on an ultra-clean bench. The isolation and culture of goat ESCs as well as the identification process followed the method proposed by Zhang et al. (2010) with simple modifications. Briefly, endometrium was prepared as small pieces of tissue and digested with type-IV collagenase (Gibco, Gaithersburg, MD, USA) for 4 h at 37 °C. The digested cell suspension was filtered through a 200-mesh cell sieve, and the filtrate was centrifuged for 6 min at 500 rpm to collect the upper suspension, followed by centrifugation for 10 min at 1200 rpm to collect the precipitated cells. Precipitated cells were resuspended with PBS and left to stand for 10 min on an ultra-clean bench, and the upper suspension was collected and centrifuged for 8 min at 1200 rpm to collect the precipitated cells. The collected precipitated cells were cultured using complete medium (89 % DMEM/F12 + 10 % fetal bovine serum + 1 % penicillin-streptomycin) (Thermo Fisher Scientific, Waltham, MA, USA) at 37 °C with 5 % CO ${}_{2}$ . All the animal experiments were approved by the Animal Ethics Committee of Guizhou University, Guiyang, China (approval no. EAE-GZU-2021-P020).

### 2.2 Detection of the proliferation and viability of ESCs in goats

The effects of different concentrations (0, 100, 200, 300 and 400 µ mol L ${}^{-1}$ ) of NAC (Xibao Biotechnology Co., Ltd., Shanghai, China) on the proliferation and viability of goat ESCs were examined using the CCK-8 method. Cells were inoculated at $5\times 10^{3}$ cells per well in 96-well plates (NEST Biotechnology Co., Ltd., Wuxi, China). After cell adherence, the cells continued to be cultured for 72 h using different concentrations of NAC medium, and the proliferation of the cells was examined at 6, 12, 24, 48 and 72 h, respectively. Ten microliters of CCK-8 solution (MCE, NJ, USA) were added to each well, and the incubation was continued for 2.5 h in a cell incubator before absorbance measurement at 450 nm using an enzyme marker (Thermo Fisher Scientific, Waltham, MA, USA).

### 2.3 SOD and glutathione reductase (GR) assays

The results of our CCK-8 assay showed that 200 µ mol L ${}^{-1}$ NAC could extremely significantly promote the proliferation of goat ESCs, so we chose a NAC concentration of 200 µ mol L ${}^{-1}$ for the subsequent assay. The cells were inoculated in 96-well plates at $5\times 10^{3}$ cells per well. After cell adherence, the culture was continued for 72 h using complete medium containing 200 µ mol L ${}^{-1}$ NAC. Cell supernatants were collected at 6, 12, 24, 48 and 72 h for SOD and GR activities, respectively. The activities of SOD and GR in the cell supernatants were assayed using a commercial kit (Hengyuan Biotechnology Co., Ltd., Shanghai, China). All operations were carried out in strict accordance with the manufacturer's instructions.

### 2.4 Total RNA extraction and qRT-PCR analysis

Cells were inoculated in six-well plates (NEST Biotechnology Co., Ltd., Wuxi, China), and after cell apposition the culture was continued for 24 h using a complete medium of 200 µ mol L ${}^{-1}$ NAC. The total RNA from goat ESCs was extracted using TRIzol^®^ reagent (Invitrogen, Grand Island, USA), and first-strand cDNA was synthesized using RT Master Mix for a qPCR II kit (MCE, NJ, USA). Changes in mRNA expression levels of endometrial receptivity marker genes (*HOXA10*, *LIF*, *PTGS2*, *PGR*) and proliferation marker genes (*PCNA*, *Cyclin D1*, *Cyclin D2*, *Cyclin E*) were detected by a CFX 9600 real-time PCR instrument (Bio-Rad, Hercules, CA, USA). The PCR primers used are shown in Table 1. For all reactions, prepare a final volume of 10 µ L of reaction mixture (containing 5 µ L 2 × RealStar Green Fast Mixture, 3 µ L ddH ${}_{2}$ O, 1 µ L cDNA, 0.5 µ L Primer Sense (10 pmol µ L ${}^{-1}$ ) and 0.5 µ L Primer Anti-sense (10 pmol µ L ${}^{-1}$ )). The reaction conditions were 95° for 2 min, 95° for 15 s, annealing (see Table 1 for details) for 30 s and 72° for 30 s. Dissolution curve analysis was performed after 40 cycles at a rate of 0.5 °C rise every 5 s from 65 to 95 °C. Three replicates were set up, and the average value was taken.

**Table 1:** Real-time PCR primer details.

Gene name	Primer sequences ( $5^{\prime }\to 3^{\prime }$ )	GenBank ID	Product size (bp)	Temperature (°C)
*HOXA10*	F: CTTCCAAAGGCGAAAACGCA R: GATCCGGTTTTCTCGGTTCA	XM_018047091.1	239	57.9
*LIF*	F: TCCCCAACAACCTGGACAAG R: ACATCAGCCGTGGCGTTC	XM_005691625.3	208	57.9
*PTGS2*	F: CTGAAAGGACTTATGGG R: AATGAGGTAAAGGGACA	XM_018060731.1	149	60
*PGR*	F: CAGCCCTATCTCAACTACCT R: TCTGCGGATTTTATCAACAA	XM_018059880.1	234	60
*PCNA*	F: GAAGAAAGTGCTGGAGGC R: TCGGAGCGAAGGGTTA	XM_005688167.3	129	60
*Cyclin E*	F: GATGTCGGCTGCTTAGAAT R: CACCACTGATACCCTGAAAC	XM_018062248.1	104	60
*Cyclin D2*	F: GAGCAGAAGTGCGAAGAGGAGG R: TTGATGGAGTTGTCGGTGTAAATG	XM_005680985.3	191	60
*Cyclin D1*	F: GCTGCGAGATGGAAAC R: AAGTAGGACACCGAGGG	XM_018043271.1	115	61.4
β *-actin*	F: TGATATTGCTGCGCTCGTGGT R: GTCAGGATGCCTCTCTTGCTC	XM_018039831.1	189	All

### 2.5 Mitochondrial membrane potential assay

The cells were inoculated in six-well plates, and after the cell growth density reached 70 %, the complete medium containing 200 µ mol L ${}^{-1}$ NAC was replaced and continued to be cultured for 24 h. Cells were digested and collected using trypsin (Thermo Fisher Scientific, Waltham, MA, USA) and washed three times with PBS. Staining was performed according to the instructions of the JC-1 kit (MCE, NJ, USA), followed by detection of cellular mitochondrial membrane potential by flow cytometry (Becton, Dickinson and Company, NJ, USA). Meanwhile, cells were inoculated on cell slides (NEST Biotechnology Co., Ltd., Wuxi, China), cultured with complete medium containing 200 µ mol L ${}^{-1}$ NAC for 24 h, washed three times with PBS, and stained using the JC-1 kit. Then the fluorescence intensity of the cells was recorded using a fluorescence microscope.

### 2.6 Statistical analysis

Differences in mRNA expression of relevant genes as well as differences in antioxidant indexes and mitochondrial membrane potential in the test and control groups were analyzed using t tests. SPSS 18.0 software (IBM Corporation, Armonk, NY, USA) was used to analyze the differences in cellular activity at different NAC concentrations over the same time period. The intensity of red fluorescence versus green fluorescence of mitochondria was analyzed using Image J software (National Institutes of Health, NY, USA). Use GraphPad 8 (GraphPad Software Inc., San Diego, CA, USA) to create histograms or line graphs. All data represent the mean ± SD. The significance threshold was set at P<0.05 . In this study, three biological replicates were performed to ensure the accuracy of the experimental data.

## 3 Results

### 3.1 Identification of ESCs in goats

**Figure 1:**
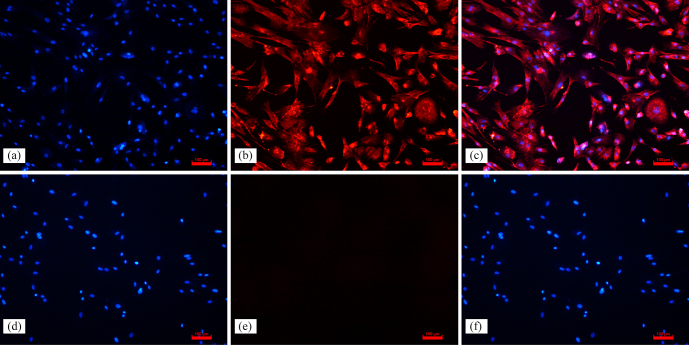
Immunofluorescence staining results (100 × ). Note: **(a, d)** ESC cell nuclear staining; **(b)** immunocytochemical staining of vimentin in ESCs; **(e)** immunocytochemical staining of keratin in ESCs; **(c)** merge **(a, b)**; **(f)** merge **(d, e)**.

In uterine tissue, keratin is only expressed in EECs, and vimentin is expressed in both EECs and ESCs (Matthews et al., 1992); therefore, identification of ESCs requires vimentin to be positive and keratin to be negative. In this experiment, indirect immunofluorescence was done on ESCs with vimentin and keratin, respectively, as primary antibodies, and it was found that more than 95 % of the cells stained with vimentin showed red fluorescence, while keratin staining did not show red fluorescence, which proved the high purity of ESCs (Fig. 1). This indicates that the primary ESCs of goats were cultured successfully.

**Figure 2:**
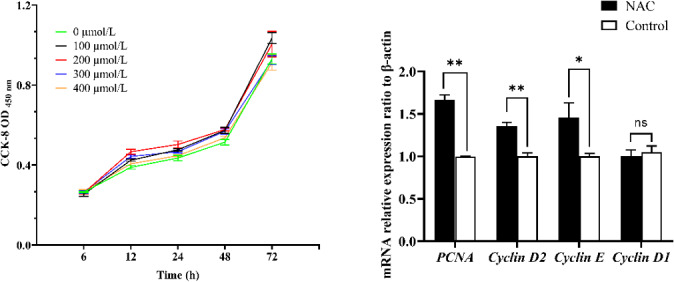
Effect of NAC on the proliferation of ESCs in goats. Note: the left panel indicates the proliferation of ESCs based on the CCK-8 assay. The right panel indicates the effect of 200 µ mol L ${}^{-1}$ NAC on the expression of proliferation-related genes in ESCs. ${}^{**}$ indicates a highly significant difference ( P<0.01 ), ${}^{*}$ indicates a significant difference ( P<0.05 ), and ns indicates a nonsignificant difference ( P>0.05 ).

### 3.2 NAC improves viability and promotes cell proliferation of goat ESCs

**Figure 3:**
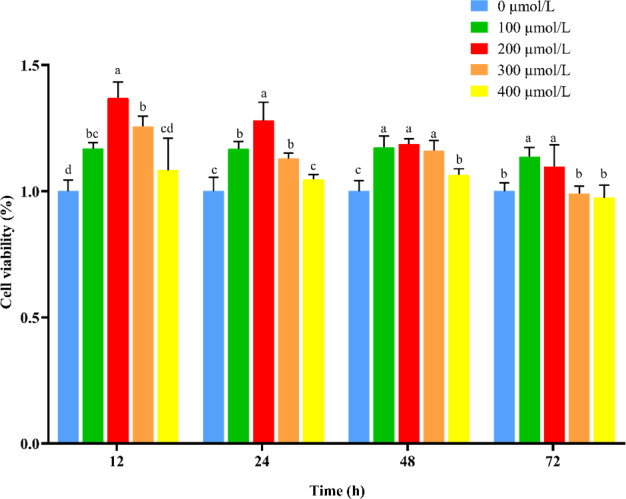
Effect of NAC on cell viability of goat ESCs. Note: different lowercase letters in the same time period indicate a significant difference ( P<0.05 ), and the same lowercase letters indicate a nonsignificant difference ( P>0.05 ).

To verify whether NAC can promote the proliferation and improve the cellular activity of goat ESCs, we performed validation using the CCK-8 and qRT-PCR methods. The results of CCK-8 showed that different concentrations of NAC promoted the proliferation of goat ESCs compared with the control group, but the most significant effect was observed at 200 µ mol L ${}^{-1}$ concentration ( P<0.01 ) (Fig. 2). The changes in mRNA expression levels of proliferation-related genes (*PCNA*, *Cyclin D1*, *Cyclin D2* and *Cyclin E*) were detected by qRT-PCR (Fig. 2), and the results showed that NAC could highly significantly promote the expressions of *PCNA* and *Cyclin D2* ( P<0.01 ) and significantly promote the expression of *Cyclin E* mRNA ( P<0.05 ), but it had no effect on the expression of *Cyclin D1* mRNA. By analyzing the cell viability, we found that the cell viability of the 100 and 400 µ mol L ${}^{-1}$ groups did not change much over time, the 100 µ mol L ${}^{-1}$ group was always higher than the control group, and the 300 and 400 µ mol L ${}^{-1}$ groups started to be lower than the control group after 72 h. However, the difference was not significant ( P>0.05 ). Cell viability was significantly higher in the 200 µ mol L ${}^{-1}$ group than in the control group at all times ( P<0.05 ) (Fig. 3).

### 3.3 NAC can improve the antioxidant activity of ESCs

**Figure 4:**
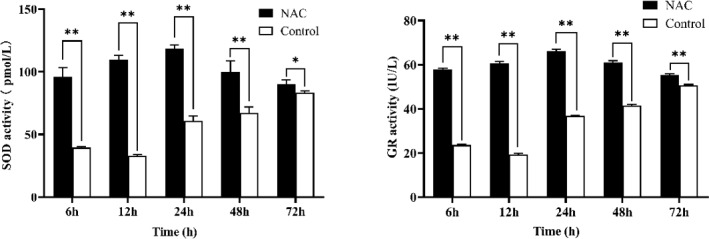
Effect of 200 µ mol L ${}^{-1}$ NAC on SOD and GR activities in goat ESCs. Note: ${}^{**}$ indicates a highly significant difference ( P<0.01 ), and ${}^{*}$ indicates a significant difference ( P<0.05 ).

NAC at 200 µ mol L ${}^{-1}$ significantly increased the activity of SOD and GR in goat ESCs at different time periods ( P<0.05 ), but both had the highest activity at 24 h (Fig. 4). In combination with the results of cell proliferation assays, all subsequent experiments were completed by culturing the cells for 24 h based on 200 µ mol L ${}^{-1}$ NAC complete medium.

### 3.4 NAC can increase the mitochondrial membrane potential of goat ESCs

**Figure 5:**
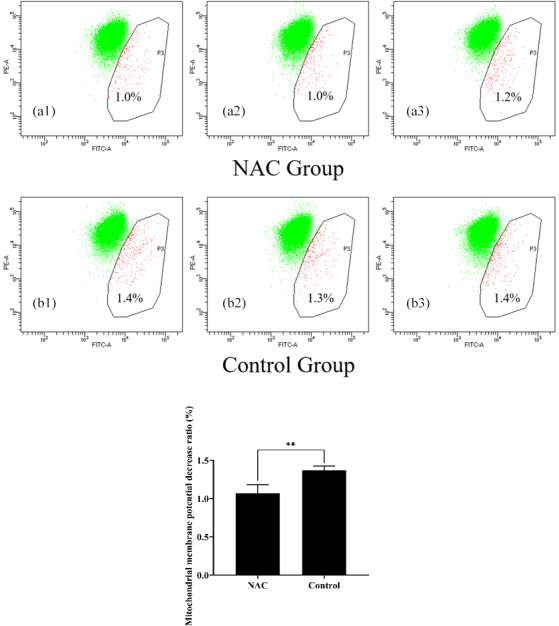
Effect of 200 µ mol L ${}^{-1}$ NAC on the mitochondrial membrane potential of goat ESCs detected by flow cytometry. Note: the percentages in Fig. 6a1, a2, a3, b1, b2 and b3 indicate the number of cells with decreased mitochondrial membrane potential as a percentage of the total number of all cells tested; Fig. 6a1, a2, a3, b1, b2 and b3 represent the three biological replicates of the NAC treatment group and the control group, respectively. ${}^{**}$ indicates a highly significant difference ( P<0.01 ).

**Figure 6:**
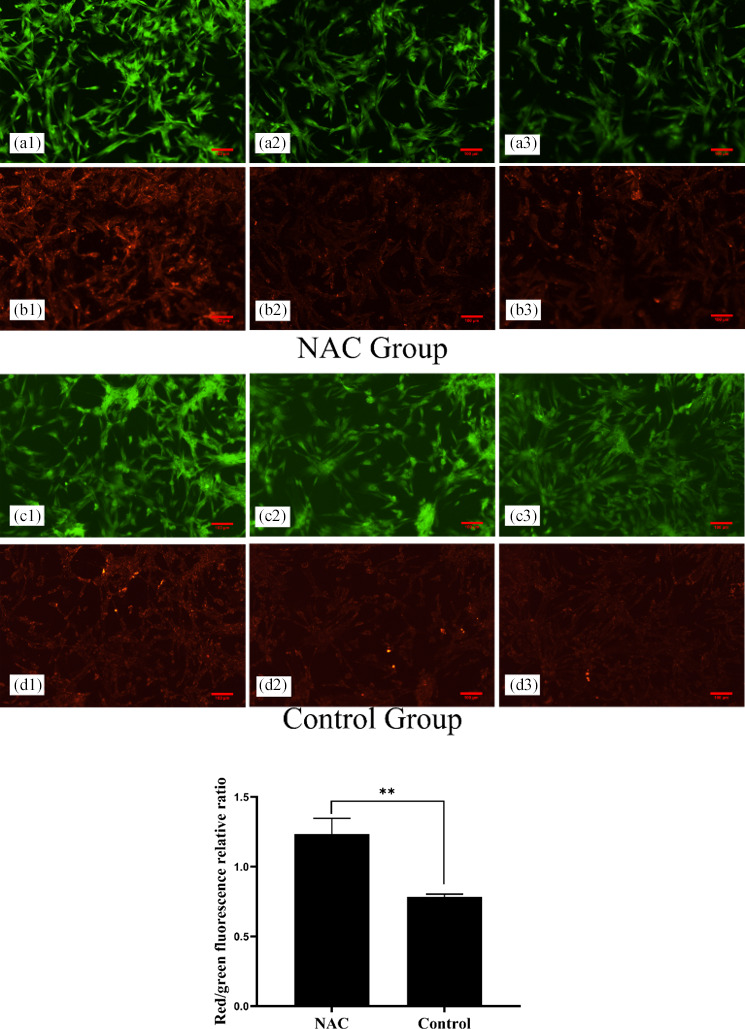
Effect of 200 µ mol L ${}^{-1}$ NAC on the mitochondrial membrane potential of goat ESCs detected by fluorescence microscopy. Note: a greater ratio of red fluorescence or green fluorescence indicates a higher cellular mitochondrial membrane potential. Panels **(a1)**, **(a2)**, **(a3)** and **(b1)**, **(b2)**, **(b3)** denote the three replicates of the NAC treatment group. Panels **(c1)**, **(c2)**, **(c3)** and **(d1)**, **(d2)**, **(d3)** denote the three replicates of the control group. ${}^{**}$ indicates a highly significant difference ( P<0.01 ).

To explore the effects of NAC on the mitochondria of goat ESCs, we examined the changes in the mitochondrial membrane potential of ESCs. Flow cytometry results showed that 200 µ mol L ${}^{-1}$ of NAC could highly significantly increase the mitochondrial membrane potential of goat ESCs (Fig. 5) ( P<0.01 ). Also, the results of our photographic analysis using fluorescence microscopy support this conclusion (Fig. 6).

### 3.5 NAC can increase the expression of endometrial receptivity marker genes

**Figure 7:**
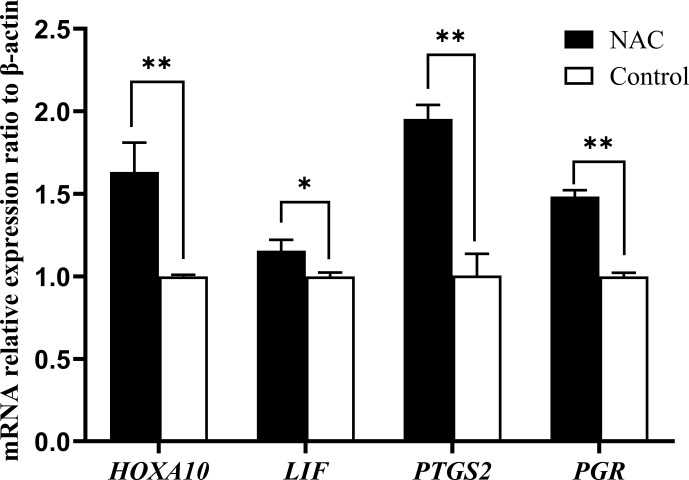
Effect of 200 µ mol L ${}^{-1}$ NAC on mRNA expression of endometrial receptivity marker genes. Note: ${}^{**}$ indicates a highly significant difference ( P<0.01 ), and ${}^{*}$ indicates a significant difference ( P<0.05 ).

We examined the mRNA expression of endometrial receptivity marker genes (*HOXA10*, *LIF*, *PTGS2* and *PGR*) using qRT-PCR. The results showed that NAC at 200 µ mol L ${}^{-1}$ could highly significantly increase the expression of *HOXA10*, *PTGS2* and *PGR* gene mRNAs ( P<0.01 ) and significantly increase the expression of *LIF* mRNA ( P<0.05 ) (Fig. 7).

## 4 Discussion

As an important place for female mammals to nurture new life, the uterus plays an essential role in the development of embryogenesis. The receptive endometrium is critical for successful implantation of the blastocyst into the uterus, which is closely related to the proliferation, differentiation and apoptosis of ESCs (Evron et al., 2011). Antioxidants were found to increase the antioxidant capacity of ESCs and improve endometrial receptivity (Qin et al., 2016). NAC, as a small-molecule antioxidant, promotes intracellular GSH biosynthesis and enhances glutathione S-transferase activity and antioxidant effects (Wu et al., 2006). In this study, we isolated ESCs from Qianbei Ma goats and successfully cultivated them through the identification of keratin and vimentin antibodies. We also used different concentrations of NAC to culture ESCs and investigate the effect of NAC on the physiological function of ESCs in goats.

Previous studies have shown that E2 and P4 acting together can promote the proliferation of ESCs and prepare for the establishment of endometrial receptivity (Zhang et al., 2013). Ma et al. (2022) found that promoting the proliferation of ESCs improved endometrial receptivity through an in vitro study. In addition, it has been shown that cell viability at the stage of blastocyst implantation is positively correlated with the successful establishment of pregnancy (Veillette et al., 2013). In this study, we cultured goat ESCs using different concentrations of NAC and found that all different concentrations of NAC promoted the proliferation of ESCs. Meanwhile, we found that NAC could significantly improve the viability of ESCs. The proliferating cell nuclear antigen (PCNA) is a proliferation marker that is widely distributed in various cells, and changes in its expression level in the cell cycle can reflect the proliferation rate of cells (Park et al., 2016). Cyclin D2 is a G1- or S-specific cell cycle protein D2 responsible for regulating the G1-to-S phase transition of the cell cycle (Pawlonka et al., 2021), Cyclin E is a key cell cycle protein that regulates cell entry into the S phase (Sauer and Lehner, 1995), and changes in the expression levels of the *Cyclin E* and *Cyclin D2* genes can reflect the proliferation of cells. Therefore, we examined the mRNA expression of these genes to verify whether NAC could promote the proliferation of ESCs in Qianbei Ma goats by altering the expression of these genes. It was found that NAC could significantly increase the expression levels of the mRNAs of these genes. This is consistent with the results of our CCK-8 assay. These findings suggest that NAC-induced cell proliferation may be related to the expression levels of *PCNA*, *Cyclin E* and *Cyclin D2*.

Oxidative stress is caused by an imbalance between ROS and protective antioxidants, affecting the entire reproductive lifespan in both men and women (Adeoye et al., 2018), and optimal levels of antioxidants can significantly ameliorate this adverse effect (Bhardwaj et al., 2021). A receptive endometrium is essential for successful pregnancy establishment in mammals, and studies have found that antioxidants are beneficial in improving endometrial receptivity (Zheng et al., 2022). Rahiminejad et al. (2016) studied 100 women and found significantly higher levels of SOD in endometrial secretions of women with persistent pregnancy compared to women with failed in vitro fertilization cycles. A sharp increase in SOD activity was observed on the day of maximum endometrial receptivity in guinea pigs (Makker et al., 2006). GR plays a central role in glutathione metabolism (Deponte, 2013) and is responsible for maintaining the supply of reduced glutathione while determining intracellular redox control or programmed cell death activation (Couto et al., 2016). Studies have shown that the activity of GR during pregnancy is significantly higher in healthy women than in women with restricted uterine growth (Aljaser et al., 2021). In this study, NAC significantly increased the activity of SOD and GR in goat ESCs, suggesting that NAC may benefit the establishment of endometrial receptivity in goats by increasing the antioxidant activity of goats.

Mitochondria regulate many cellular functions, including mitochondrial ROS production (Hekimi et al., 2016), regulation of apoptosis (Yee et al., 2014), activation of the endoplasmic reticulum stress response (Kim et al., 2016) and adenosine triphosphate (ATP) production (van der Bliek et al., 2017). Mitochondria are the main organelles involved in apoptosis and control of apoptotic pathways in cells and the mitochondrial membrane potential is important for maintaining normal physiological functions of mitochondria, while a decrease in mitochondrial membrane potential leads to early apoptosis and a rise in ROS (Zaib et al., 2022). To verify whether NAC can increase the mitochondrial membrane potential of goat ESCs, we used NAC to culture goat ESCs for 24 h and then examined the mitochondrial membrane potential of ESCs using flow cytometry and fluorescence microscopy. The results showed that NAC could increase the mitochondrial membrane potential of goat ESCs extremely significantly. This implies that NAC may inhibit early apoptosis as well as the accumulation of ROS by improving the mitochondrial membrane potential of goat ESCs.

Extensive evidence suggests that *HOXA10*, *LIF*, *PTGS2* and *PGR* gene expressions are positively associated with the establishment of mammalian endometrial receptivity and that they are important factors in the regulation of endometrial receptivity and embryo implantation (Das, 2010; Cha et al., 2012; Wetendorf and DeMayo, 2014; Mrozikiewicz et al., 2021). There are lower expression levels of *PGR*, *PTGS2*, *LIF* and *HOXA10* genes in women with impaired endometrial receptivity, and there is higher expression in women with normal endometrial receptivity (Achache et al., 2010; Paramonova et al., 2018; Tan et al., 2021). In this study, we determined that NAC significantly increased the relative expressions of *HOXA10*, *LIF*, *PTGS2* and *PGR* gene mRNAs in goat ESCs. Therefore, we hypothesize that NAC may enhance the endometrial receptivity of goats by promoting the expression of these genes, thereby improving the rate of embryo attachment and maintaining normal establishment of early pregnancy in goats.

## 5 Conclusions

This study shows that NAC, as a small-molecule antioxidant, may improve endometrial receptivity in goats by promoting the proliferation of ESCs and increasing the viability, antioxidant activity, mitochondrial membrane potential and expression of endometrial receptivity marker genes, thereby directly or indirectly affecting the implantation and pregnancy maintenance of goat blastocysts.

## Data Availability

The raw data supporting the conclusions of this article will be made available by the authors upon request, without undue reservation.
